# Supplementary material for: Direct Photochemical Synthesis of Substituted Benzo[b]fluorenes
Source: Org Lett. 2024 Nov 25;26(48):10364–8. doi: 10.1021/acs.orglett.4c03978 (PMC11629382; doi:10.1021/acs.orglett.4c03978)
Supplement: Supplementary file 2 — ol4c03978_si_002.zip [file ol4c03978_si_002.zip › FID for Publication/5d/20240424_RC-2-C-5-69_01/plots/RC-2-C-5-69_FLUORINE_20240425_01_plot01.pdf]

RC-2-C-5-69

Location	3	Pulse sequence	FLUORINE	Temperature	25	Suboperator	RuairiCrawford
Date collected	2024-04-25	Solvent	dmso	Spectrometer	Agilent-NMR-vnmrs500	Operator	MBaumann

SAMPLE

date	Apr 25 2024
solvent	dmso
file	/home/data/MBaumann/newucd500/RuairiCrawford/2024/20240424_RC-2-C-5-69_01/RC-2-C-5-69_FLUORINE_20240425_01.fid

ACQUISITION

sw	131578.9
at	1.000
np	263158
fb	47800
bs	2
ss	2
d1	1.000
nt	16
ct	16

TRANSMITTER

tn	F19
sfrq	470.449
tof	712.0
tpwr	58
pw	6.600

DECOUPLER

dn	C13
dof	0
dm	nnn
decwave	W40_One_NMR_new
dpwr	39
dmf	32258

PRESATURATION

satmode	n
wet	n

SPECIAL

temp	25.0
gain	60
spin	0
hst	0.008
pw90	9.900
alfa	10.000

FLAGS

il	n
in	n
dp	y
hs	nn

PROCESSING

fn	131072

DISPLAY

sp	-117427.1
wp	131578.9
rfl	117427.1
rfp	0
rp	72.4
lp	0

PLOT

wc	252
sc	8
vs	6
th	0
ai cdc ph	

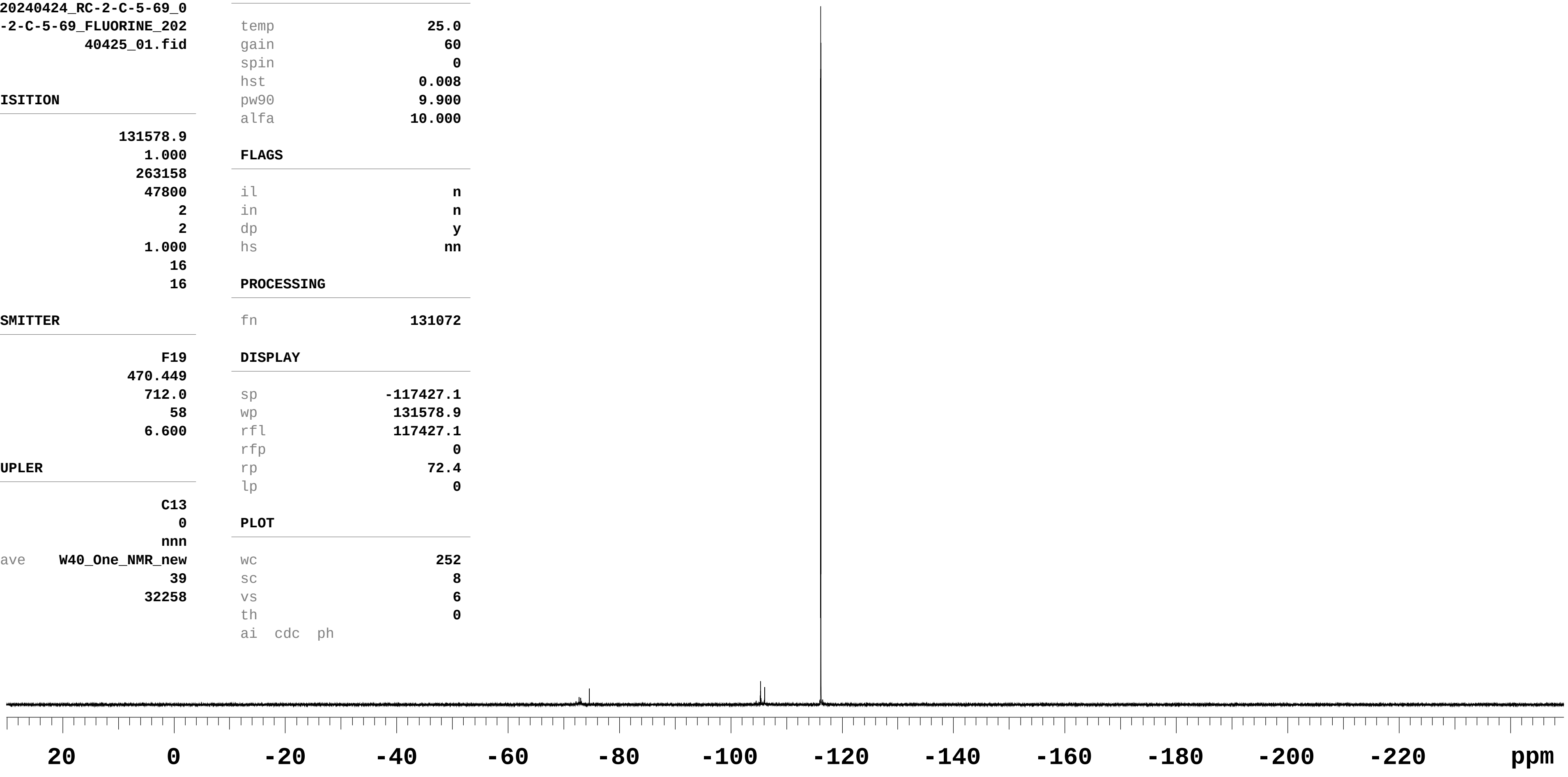

Plotname: RC-2-C-5-69_FLUORINE_20240425_01_plot01

Data file /home/data/MBaumann/newucd500/RuairiCrawford/2024/20240424_RC-2-C-5-69_01/RC-2-C-5-69_FLUORINE_20240425_01.fid

Plot date 2024-04-25
